# Associations between lifestyle factors and multidimensional frailty: a cross-sectional study among community-dwelling older people

**DOI:** 10.1186/s12877-021-02704-x

**Published:** 2022-01-03

**Authors:** Marcel A. L. M. van Assen, Judith H. M. Helmink, Robbert J. J. Gobbens

**Affiliations:** 1 Faculty of Social and Behavioral Sciences, Tilburg University, Tilburg, the Netherlands; 2 Faculty of Social and Behavioural Sciences, Utrecht University, Utrecht, the Netherlands; 3 GGD Hart voor Brabant, ‘s-Hertogenbosch, the Netherlands; 4 Faculty of Health, Sports and Social Work, Inholland University of Applied Sciences, De Boelelaan 1109, 1081 HV Amsterdam, the Netherlands; 5 Zonnehuisgroep Amstelland, Amstelveen, the Netherlands; 6 Department Family Medicine and Population Health, Faculty of Medicine and Health Sciences, University of Antwerp, Antwerp, Belgium

**Keywords:** older people, frailty, lifestyle, Tilburg Frailty Indicator

## Abstract

**Background:**

Multidimensional frailty, including physical, psychological, and social components, is associated to disability, lower quality of life, increased healthcare utilization, and mortality. In order to prevent or delay frailty, more knowledge of its determinants is necessary; one of these determinants is lifestyle. The aim of this study is to determine the association between lifestyle factors smoking, alcohol use, nutrition, physical activity, and multidimensional frailty.

**Methods:**

This cross-sectional study was conducted in two samples comprising in total 45,336 Dutch community-dwelling individuals aged 65 years or older. These samples completed a questionnaire including questions about smoking, alcohol use, physical activity, sociodemographic factors (both samples), and nutrition (one sample). Multidimensional frailty was assessed with the Tilburg Frailty Indicator (TFI).

**Results:**

Higher alcohol consumption, physical activity, healthy nutrition, and less smoking were associated with less total, physical, psychological and social frailty after controlling for effects of other lifestyle factors and sociodemographic characteristics of the participants (age, gender, marital status, education, income). Effects of physical activity on total and physical frailty were up to considerable, whereas the effects of other lifestyle factors on frailty were small.

**Conclusions:**

The four lifestyle factors were not only associated with physical frailty but also with psychological and social frailty. The different associations of frailty domains with lifestyle factors emphasize the importance of assessing frailty broadly and thus to pay attention to the multidimensional nature of this concept. The findings offer healthcare professionals starting points for interventions with the purpose to prevent or delay the onset of frailty, so community-dwelling older people have the possibility to aging in place accompanied by a good quality of life.

## Introduction

Frailty is a concept that is in the spotlight of science and practice. This is not surprising because frailty is closely related to ageing [1] and population aging is occurring all over the world [2]. The debate about the definition of frailty, conceptual as well as operational, is still ongoing. Two approaches can be distinguished; one approach considers frailty as a biological concept and includes only physical problems that older people may have in the definition. The conceptual definition of Fried et al. [3] is a very good example of this definition: “frailty is a biologic syndrome of decreased reserve and resistance to stressors, resulting from cumulative declines across multiple physiologic systems, causing vulnerability to adverse outcomes”. The corresponding operational definition, the phenotype of frailty, also shows this. According to this phenotype, an older individual is frail if he/she meets at least three of the following five criteria: unintentional weight loss, weakness, poor self-reported endurance, slow walking speed, and low physical activity [3]. The other approach to frailty starts from a holistic view of mankind and, in addition to the physical domain, also includes other domains such as the psychological and social domains. The definition developed by Gobbens et al. [4] fits well with this latter approach: frailty is a dynamic state affecting an individual who experiences losses in one or more domains of human functioning (physical, psychological, social), which increases the risk of adverse outcomes, and is influenced by many variables.

Many studies have shown that frailty is related to adverse outcomes in community-dwelling older people such as disability [5], lower quality of life [6], hospitalization [7], institutionalization [8], and mortality [9]. Therefore, it is important to identify older people at risk for frailty at an early stage, with the aim to prevent or delay frailty and subsequently also the adverse outcomes of frailty [10, 11]. In order to identify these people, knowledge of the determinants of frailty is necessary. Well-known determinants of frailty are greater age [1, 12], gender (being a woman) [1, 13], lower education [14], low income [13, 15], and living without a partner [13, 14]. Another important determinant of frailty is having an unhealthy lifestyle [16–19]. An unhealthy lifestyle is characterized by smoking, excessive alcohol use, poor dietary habits, and low physical activity. While healthcare professionals can hardly intervene in the aforementioned other determinants, in the case of lifestyle this is different. This makes the examination of the effect of lifestyle factors on frailty even more urgent.

For each of the four lifestyle factors smoking, excessive alcohol use, poor dietary habits, and low physical activity, systematic reviews and/or meta-analyses, have been carried out on their effects on frailty. A systematic review conducted by Kojima et al. [20] found that four of five included studies showed that baseline smoking was associated with developing or worsening frailty at follow-up among community-dwelling older people. However, it is relevant to note that most studies did not control or very limitedly for confounding factors as age, gender, and education, which prevents drawing conclusions on causal relationships (e.g., a confounding variable may, on average, yield more smoking and lower frailty). A recent longitudinal study among 2542 community-dwelling older people aged ≥60 years in England also demonstrated that current smokers were more frail than non-smokers; smoking was associated with incident frailty in a 4-year follow-up, controlled for confounders (e.g. age, gender, and socioeconomic status) [21].

With regard to the associations between alcohol use and frailty, the results of cohort studies are mixed [22–24]. Ortola et al. [22] concluded that certain drinking patterns, including drinking only with meals and moderate alcohol use, were associated with a lower risk of frailty in community-dwelling older people (≥60 years). This finding is supported by Kojima et al. [24], who concluded that incident risk of frailty was lower at around 15 g. alcohol on a daily basis, but was higher for higher intakes of alcohol. Furthermore, the Lausanne cohort study among 1,564 persons aged 65–70 years revealed that non-alcohol users had two-times higher odds of prevalent and 3-year incident frailty than light-to-moderate alcohol users, after controlling for poorer heath status at baseline [23].

Two systematic reviews were conducted aiming to determine the associations between nutrition and frailty. Lorenzo-Lopez et al. [25] included four and three studies in their review confirming that higher protein intake was associated with a lower frailty risk and that nutrient quality is inversely associated with the risk of frailty, respectively. The aim of the systematic review and meta-analysis by Wang et al. [26] was to summarize the effect of adherence to a Mediterranean diet on frailty. They included five prospective studies and one cross-sectional study showing that a Mediterranean diet is associated with a lower risk of frailty. A more recent narrative review found that most identified studies found that low protein intake is associated with physical frailty, with regard to both prevalence and incidence [27]; unfortunately, this review does not cover confounders. This research group recommends carrying out future studies examining the associations between dietary protein and multidimensional frailty, including also psychological and social limitations that older people may have, e.g., mood, cognition.

Concerning physical activity, evidence suggests that more than 85% of the older people are not sufficiently active enough to meet the recommendations by the World Health Organization (WHO) of 150 minutes of moderate to vigorous physical activity within a week; older people spend around 10 hours per waking day sedentary [28]. The systematic review carried out by Tolley et al. aimed to synthesize the available evidence with regard to the associations of objectively measured habitual physical activity and frailty in community-dwelling older people. This review, based on 23 articles totaling 7,696 participants, concludes that objective measures of physical activity are associated with frailty, regardless the operational definition of frailty [29]. Based on four studies, a meta-analysis also showed that physical activity probably prevents frailty [30].

In many of the aforementioned studies regarding lifestyle factors, physical frailty, commonly represented by the phenotype of frailty [3], was the outcome measure, disregarding other domains of frailty (psychological and social). To a lesser extent, a multidimensional outcome measure of frailty was used such as the Frailty Index [19, 31]. Our study distinguishes itself from previous studies, because we aimed to determine the associations between smoking, alcohol use, nutrition, physical activity, and the physical, psychological and social domains of frailty separately in a large sample of community-dwelling older people. While examining these associations, we employ a much larger sample of older people (>45,000) than previous studies, which was also meant to be representative. Because of this large sample, we are sure to even detect small effects (ie, statistical power to detect a small effect approaches 1), and we can focus on assessing effect sizes of lifestyle factors on frailty. Finally, we controlled for the effect of confounders (age, gender, marital status, education, and household income) to exclude some alternative explanations of associations.

## Methods

### Study population and data collection

The data in this study were collected in 2012 as part of a general health questionnaire of the Public Health Services in the Netherlands. Samples of community-dwelling older people aged 65 years or older were randomly drawn by Statistics Netherlands from the registers of the municipalities in the provinces Zeeland and Noord-Brabant (small cities and rural areas), including around 381,000 and 2,470,000 inhabitants. As described in a previous study [13], the following exclusion criteria were used: older persons residing in an institution, prisoners, older people staying in refugee asylum centers, participation in other research by Statistics Netherlands. Moreover, up to one older person per household was included in the sample.

In total 77,102 older people were invited by letter to fill in a questionnaire on paper or on the internet; these individuals received a reminder twice. Of these people, 45,336 participated, corresponding to a response rate of 58.8%. The sample has two parts, one smaller part (N = 10,421, with residents of West-Brabant) (sample WB) that completed all questions and one larger part (N = 34,915, with residents of Hart voor Brabant, Brabant Zuidoost and Zeeland) (sample HZ) that did not receive the questions on nutrition. The complete data set was also used to construct norm scores of frailty as a function of demographic characteristics [13].

### Measurements

The questionnaire contained questions regarding sociodemographic characteristics, lifestyle factors, physical and psychological health, chronic diseases, falls, well-being, frailty, and health care utilization. For the purpose of the present study, we only used the data on sociodemographic characteristics, lifestyle factors, and frailty.

#### Frailty

We used the Tilburg Frailty Indicator (TFI) part B for assessing frailty. The TFI is a self-report questionnaire to identify multidimensional frailty; frailty is observed in three domains: physical, psychological, and social [32]. The physical domain includes eight components: physically unhealthy, unintentional weight loss, difficulty in walking, difficulty in maintaining balance, poor hearing, poor vision, lack of strength in the hands, and physical tiredness. The psychological domain of the TFI contains four components: problems with memory, feeling down, feeling nervous or anxious, and unable to cope with problems. Finally, the social domain includes the following three components: living alone, lack of social relations, and lack of social support. The maximum score for total frailty and the physical, psychological and social domains of frailty is 15, 8, 4, and 3 points, respectively, with higher scores corresponding to more frailty. The TFI was developed in the Netherland and has shown good psychometric properties among Dutch community-dwelling older individuals [32, 33]. The TFI was then translated into other languages and validated in countries around the world, including Poland, Jordan, Turkey, and China [34–37].

#### Lifestyle factors

We assessed four lifestyle factors: smoking, use of alcohol, nutrition, and physical activity, using the participants’ answers to the questionnaire. Smoking was operationalized with two variables, “smoking past” (“Did you smoke in the past?”, 0 = no, 1 = yes), and “smoking” (“How many cigarettes do you smoke on average per day?”, subjects enter a number).

Use of alcohol was assessed in a similar way with the variables “drinking past” (“I never drank alcohol”, 0 = no [never], 1 = yes [ever]) and “drinking”. The variable “drinking” was created by adding the scores of “number of days drinking in the weekend” (0, 0.5 (“less than one day”), 1, 2, 3) to “number of days drinking on weekdays” (0, 0.5 (“less than one day”), 1, 2, 3, 4), resulting in a scale from 0 to 7.

Lifestyle factor nutrition was assessed with four variables “fruits”, “vegetables”, “hot meal”, “breakfast”, which were weekly correlated (strongest correlation of 0.28 between breakfast and hot meal). For these variables, we asked “How many days a week … ”: “do you eat fruit or drink a glass of juice?” (fruits), “do you eat vegetables? (vegetables in casseroles also count, but a lettuce leaf on a sandwich, for example, does not count)” (vegetables), “do you eat a hot meal?” (hot meal), and “do you eat breakfast? (breakfast-drink, breakfast bar, muesli and the like, also count as breakfast)” (breakfast). For each nutrition variable this resulted in a scale from 0 to 7.

With respect to lifestyle factor physical activity, participants were asked to answer the question, “Consider a normal week in the last months. Can you indicate how many days per day you performed *X* and how much time on average you spent on *X* on such a day?”. Subjects entered the number of days, hours, and minutes, which was transformed into one measure equal to the average number of minutes per week spent on *X*. Activities *X* were “walk work” (walking to school or work), “cycle work” (cycling to school or work), “active work Light” (light or moderate physical activity at work or school), “active work Heavy” (heavy physical activity at work or school), “housework Light” (light or moderate housework), “housework Heavy” (heavy housework), leisure activities “walking”, “cycling”, “gardening”, “do-it-yourself”, and “sports”. As participants could specify four sports, “sports” was the sum of the times spent on up to four sports. As the correlations between the activities were generally weak (33 [60%], 17 [31%], 3 [5.4%], 2 [3.6%] in intervals (–0.1, 0.1), (0.1, 0.2), (0.2, 0.3), respectively), we could not create a physical activity scale and decided to assess the effects of these individual activities on frailty.

Probably because of the rather complex response format (requiring the completing of days, hours, and minutes spent on each activity separately), some participants entered answers that were deemed implausible. We decided to exclude participants with implausible response patterns in the correlation and regression analyses, that is, patterns where for at least one of activities (i) at least 12 hours per day was spent on the activity each day (or 5 hours for one sports activity), or (ii) more than 300 minutes per day, except for homework where we considered more than 480 minutes per day implausible, or (iii) at least 16 hours per day summed across all activities. For these reasons, we excluded 980 (9.4%) and 3195 (9.2%) of response patterns in both samples in the correlation and regression analyses. Finally, because all activities included high outlying scores, we transformed all activities by log(*X*+1), with *X* denoting the time spent on an activity in minutes, to reduce their impact on the estimated regression equation.

#### Sociodemographic characteristics

The sociodemographic characteristics of the participants considered were age, gender, marital status, education (“What is the highest level of education you have completed?”), and household income (“Over the past 12 months, have you struggled to get along with your household's income?”). Age was centered at mean age (*M* = 73.6) and divided by 5, and squared age was also considered as it is known that frailty increases at an increasing rate as a function of age [13]. We refer to Table 1 for a description of the response categories, or Van Assen et al. [13] for a detailed explanation and motivation of the coding of these variables.

**Table 1:** Participant characteristics (N = 45,336)

	Sample 1: West-Brabant (WB)		Sample 2: Hart voor Brabant, Brabant Zuidoost, Zeeland (HZ)	
	(N=10,421)	Missings	(N=34,915)	Missings
**Background characteristics**				
*Age, mean ±SD, range*	73.0 ± 6.5, 65–100	124	73.5 ± 6.6, 65–103	323
*Gender, % of women*	5454 (52.3)	-	18,216 (52.2)	-
*Marital status*		371		1000
Married/cohabiting	6961 (69.3)		23,200 (68.4)	
Not married	284 (2.8)		1205 (3.6)	
Divorced	435 (4.3)		1794 (5.2)	
Widowed	2370 (23.6)		7716 (22.8)	
*Education*		617		1997
Low	2104 (21.5		5835 (17.7)	
Middle-low	4775 (48.7)		15,228 (46.3)	
Middle-high	1552 (15.8)		5885 (17.9)	
High	1373 (14.0)		5970 (18.1)	
*Household income*		494		1678
No, don’t bother	4455 (42.8)		16,323 (49.1)	
No, don't bother, but I have to keep an eye on my expenses	4190 (42.2)		13,011 (39.1)	
Yes, some difficulty	1048 (10.6)		3188 (9.6)	
Yes, great difficulty	234 (2.4)		715 (2.2)	
*Frailty, mean ±SD, range*				
Total	2.67 (2.86)	2791	2.61 (2.81), 0–14	9096
Physical	1.37 (1.84)	1916	1.34 (1.82), 0–8	6071
Psychological	0.83 (1.07)	974	0.80 (1.06), 0–4	3116
Social	0.59 (0.81)	1576	0.58 (0.79), 0–3	5042
**Lifestyle factors**				
*Smoking*				
Smoking past, % of Yes	6445 (65.7)	608	21,694 (66.4)	2241
Smoking (cigarettes a day), mean ±SD, range	1.41		1.31	
*Alcohol use*				
Drinking past, % of Yes	8469 (85.4)	503	29,138 (87.1)	1450
Drinking (days a week), mean ±SD, range	2.85 (2.78), 0–7	961	3.03 (2.81), 0–7	3000
*Nutrition (days a week), mean ±SD, range*				
Fruits	4.24 (1.89), 0–7	1883	-	
Vegetables	2.64 (1.92), 0–7	1648	-	
Hot meal	5.66 (1.21), 0–7	852	-	
Breakfast	6.58 (1.44), 0–7	808	-	
*Physical activity (log of number of minutes a week), mean ±SD, range*				
Walk work	0.62 (1.43), 0–7.45	2545	0.40 (1.18), 0–7.83	7514
Cycle work	0.54 (1.32), 0–7.65	2545	0.40 (1.15), 0–7.65	7514
Housework Light	3.45 (2.05), 0–8.20	1031	3.43 (1.09), 0–8.48	3839
Housework Heavy	1.25 (1.62), 0–7.83	1031	1.19 (1.60), 0–8.48	3839
Active work Light	1.30 (2.09), 0–6.74	3145	1.03 (1.95), 0–6.75	8847
Active work Heavy	0.57 (1.37), 0–6.74	3145	0.41 (1.20), 0–6.74	8847
Walking	2.02 (1.81), 0–8.19	598	2.07 (1.79) 0–8.60	1820
Cycling	1.89 (1.85), 0–7.50	598	1.92 (1.86) 0–7.75	1820
Gardening	1.62 (1.82), 0–7.65	598	1.63 (1.79), 0–7.65	1820
Do–it–yourself	1.11 (1.73), 0–7.63	598	1.13 (1.73), 0–8.32	1820
Sports	1.11 (1.70), 0–7.41	598	1.23 (1.73), 0–8.77	1820

SD = Standard deviation

### Statistical analyses

After excluding the data of implausible response patterns, we calculated the descriptive statistics (mean, SD, range, or frequency and percentage, and percentage of missing values) for the samples WB and HZ.

After computing the (Pearson) correlations of all lifestyle factors with frailty (total, physical, psychological, social) for the data of both samples combined, we carried out two sets of multiple regression analyses. The first set of analyses regressed frailty on lifestyle factors smoking, use of alcohol, and physical activity for data of both samples combined. We combined the two samples because the effects of all predictors on each of the frailty domains were indistinguishable across both samples (increased explained variance at most .001, all *p*-values > .044). The second set also included lifestyle factor nutrition but was only ran on data of the WB sample as no data on nutrition were collected for the larger HZ sample. In each set, we carried out two analyses. In the first analysis, we established the increase in explained variance (*ΔR*^*2*^) of a lifestyle factor (smoking, use of alcohol, physical activity, and also nutrition for the second sample) after controlling for the effects of the demographic variables. In the second analysis, we established how much a lifestyle factor contributed to the explanation (*ΔR*^*2*^) after controlling for all other predictors (demographic as well as lifestyle). We calculated and interpreted effect sizes of a lifestyle factor using Cohen’s *f*^2^ [38]. In line with Cohen [38] and with Brydges [39], *f*^*2*^ ≤ .02 and *r* ≤ 0.1 was considered a small effect size. For each individual predictor, we also presented the estimates and test results of the full model, that is, the model that includes all predictors.

All analyses were run using SPSS 24. As our sample size was so large, we considered p < .001 as statistically significant, but focus on effect size for interpretation.

## Results

### Participants

Table 1 shows the scores (mean, SD or proportions, and number of missing values) for all variables in both samples. The average age was about 73 years in both samples, and the percentage of women slightly above 52%. The two samples were similar with respect to demographic characteristics, although the second sample had relatively more highly educated participants (18.1 vs. 14%), more participants with a supposedly sufficient or high income (49.1 vs. 42.8%). The second sample also showed a higher mean and SD on drinking.

### Correlations and regression analyses

The correlations between frailty domains were 0.450, 0.292, 0.364 (p<0.001), between physical and psychological, physical and social, and psychological and social, respectively. All lifestyle factors were associated with total frailty (see Table 2), where older people were on average less frail when they smoked in the past, smoked less, drank and drank in the past, had more healthy nutrition (intake of fruit and vegetables, and having regular breakfast and dinner), and had more physical activity. Associations with total frailty were either small (*r* <0.1), or somewhat larger, such as with both drinking variables, a hot meal and intake of vegetables, and all physical activity variables except walking and cycling to work (0.1< *r* <0.3). Associations of lifestyle factors with frailty domains were similar, except that walking and cycling to work were not associated to social frailty.

**Table 2:** Correlations between lifestyle factors and frailty total and frailty domains

	Frailty total	Frailty physical	Frailty psychological	Frailty social
**Lifestyle factors**				
*Smoking*				
Smoking past	–0.042***	–0.022***	–0.031***	–0.058***
Smoking	0.061***	0.036***	0.043***	0.072***
*Use of alcohol*				
Drinking past	–0.162***	–0.158***	–0.090***	–0.101***
Drinking	–0.202***	–0.197***	–0.120***	–0.1389***
*Nutrition‡*				
Fruits	–0.027*	–0.019	–0.028*	–0.019
Vegetables	–0.100***	–0.086***	–0.068***	–0.068***
Hot meal	–0.141***	–0.103***	–0.104***	–0.157***
Breakfast	–0.055***	–0.038***	–0.048***	–0.053***
*Physical activity*				
Walk work	–0.050***	–0.063***	–0.020***	0.010
Cycle work	–0.074***	–0.090***	–0.037***	–0.013*
Homework Light	–0.148***	–0.192***	–0.070***	–0.023***
Homework Heavy	–0.235***	–0.272***	–0.105***	–0.088***
Active work Light	–0.105***	–0.101***	–0.064***	–0.057***
Active work Heavy	–0.122***	–0.124***	–0.071***	–0.063***
Walking	–0.195***	–0.212***	–0.089***	–0.090***
Cycling	–0.292***	–0.294***	–0.159***	–0.178***
Gardening	–0.261***	–0.264***	–0.155***	–0.139***
Do-it-yourself	–0.238***	–0.204***	–0.159***	–0.173***
Sports	–0.171***	–0.170***	–0.099***	–0.104***

*‡* Correlations (pair-wise exclusion of missing values) with nutrition are only based on data of sample WB, whereas other correlations are based on the data of both samples together.

* * p<0.05; ***p<0.001

Turning to the results of the regression analyses predicting total frailty for the entire sample (second column, Table 3), 23% of the variance of total frailty was explained by background variables. More frailty was associated with higher age, with being a woman, lower education, and struggling to get along with household's income. Controlling for demographic variables, explained variance of total frailty increased 0.7% by the smoking variables (Cohen’s *f*^*2*^ = 0.009), 0.8% by the alcohol variables (Cohen’s *f*^*2*^ = 0.01), and 6.5% by the physical activity variables (Cohen’s *f*^*2*^ = 0.095). When controlling for the effects of all predictors, the effects of smoking, drinking, and physical activity variables reduced somewhat to 0.4%, 0.5%, and 5.6%, of increased explained variance, respectively. In total, all predictors together explained 32.1% of total frailty. In this final model with all predictors, the associations controlled for other predictors’ effects were such that less total frailty was associated with lower scores on smoking variables, higher scores on the drinking variables, and higher scores on the physical activity variables (except active work Light and active work Heavy).

**Table 3:** Results of regression analyses: Effect of background characteristics and lifestyle factors (smoking, use of alcohol, physical activity, nutrition) on frailty total and domains^1^

	Frailty total*‡*			Frailty physical			Frailty psychological			Frailty social*‡*		
	B	SE	*p*	B	SE	*p*	B	SE	*p*	B	SE	*p*
**Background characteristics**												
Age_c	0.533	0.015	<0.001	0.316	0.010	<0.001	0.079	0.006	<0.001	0.135	0.004	<0.001
Age2_c	0.082	0.008	<0.001	0.048	0.005	<0.001	0.014	0.003	<0.001	0.019	0.002	<0.001
Gender (women)	0.703	0.043	<0.001	0.237	0.027	<0.001	0.268	0.017	<0.001	0.203	0.012	<0.001
Not married*‡*				0.002	0.073	0.976	–0.004	0.047	0.937			
Divorced*				0.053	0.058	0.365	0.038	0.037	0.301			
Widowed*				0.298	0.095	<0.001	0.141	0.031	<0.001			
Education	–0.080	0.019	<0.001	–0.054	0.012	<0.001	–0.031	0.007	<0.001	0.005	0.005	0.400
Income	0.750	0.023	<0.001	0.426	0.015	<0.001	0.215	0.009	<0.001	0.127	0.007	<0.001
*ΔR*^*2*^	0.230 F(5, 28295) = 1699.94, p<0.001			0.205 F(8, 31477) = 1016.119, p <0.001			0.088 F(8, 34620) = 416.15, p <0.001			0.128 F(5, 32725) = 958.84, p <0.001		
**Lifestyle factors**												
*Smoking*												
Smoking past	0.197	0.039	<0.001	0.128	0.024	<0.001	0.040	0.015	0.010	0.024	0.011	0.037
Smoking	0.029	0.004	<0.001	0.006	0.002	0.013	0.008	0.001	<0.001	0.014	0.001	<0.001
*ΔR*^*2*^	0.007 F(2, 26915) = 118.94, p <0.001			0.003 F(2, 29728) = 60.86, p <0.001			0.002 F(2, 32528) = 42.99, p <0.001			0.008 F(2, 30850) = 149.71, p <0.001		
	0.004 F(2,20564) = 54.30, p <0.001			0.001 F(2,22091) = 19.92, p <0.001			0.002 F(2, 23299) = 22.28, p <0.001			0.007 F(2, 22817) = 98.83, p <0.001		
*Use of alcohol*												
Drinking past	–0.195	0.059	<0.001	–0.222	0.037	<0.001	0.010	0.023	0.653	0.008	0.017	0.646
Drinking	–0.070	0.007	<0.001	–0.044	0.004	<0.001	–0.013	0.003	<0.001	–0.017	0.002	<0.001
*ΔR*^*2*^	0.008 F(2, 27170) = 140.56, p <0.001			0.010 F(2,29893) = 192.78, p <0.001			0.001 F(2, 32506) = 21.91, p <0.001			0.002 F(2, 30996) = 39.40, p <0.001		
	0.005 F(2, 20564) = 78.41, p <0.001			0.006 F(2,22091) = 104.05, p <0.001			0.001 F(2, 23299) = 13.08, p <0.001			0.003 F(2, 22817) = 40.36, p <0.001		
*Physical activity*												
Walk work	–0.044	0.018	0.016	–0.052	0.011	<0.001	–0.002	0.007	0.746	0.013	0.005	0.011
Cycle work	–0.071	0.019	<0.001	–0.061	0.012	<0.001	–0.020	0.007	0.006	0.008	0.006	0.152
Homework Light	–0.067	0.010	<0.001	–0.052	0.006	<0.001	–0.025	0.004	<0.001	0.010	0.003	<0.001
Homework Heavy	–0.111	0.012	<0.001	–0.110	0.008	<0.001	–0.005	0.005	0.295	0.004	0.004	0.264
Active work Light	0.003	0.011	0.807	0.003	0.007	0.665	0.002	0.004	0.719	0.000	0.003	0.941
Active work Heavy	–0.019	0.018	0.285	–0.014	0.011	0.209	–0.007	0.007	0.289	–0.001	0.005	0.783
Walking	–0.116	0.010	<0.001	–0.095	0.006	<0.001	–0.010	0.004	0.012	–0.005	0.003	0.071
Cycling	–0.182	0.010	<0.001	–0.119	0.006	<0.001	–0.036	0.004	<0.001	–0.027	0.003	<0.001
Gardening	–0.137	0.011	<0.001	–0.100	0.006	<0.001	–0.026	0.004	<0.001	–0.009	0.003	0.003
Do-it-yourself	–0.065	0.012	<0.001	–0.023	0.007	<0.001	–0.023	0.005	<0.001	–0.017	0.003	<0.001
Sports	–0.065	0.009	<0.001	–0.037	0.007	<0.001	–0.018	0.004	<0.001	–0.009	0.003	0.005
*ΔR*^*2*^	0.065 F(11,22008) = 189.76, p <0.001			0.083 F(11,23743) = 257.53, p <0.001			0.018 F(11, 25173) = 46.92, p <0.001			0.010 F(11,24595) = 25.70, p <0.001		
	0.056 F(11,20564) = 153.79, p <0.001			0.074 F(11,22091) = 217.22, p <0.001			0.016 F(11, 23299) = 38.48, p <0.001			0.007 F(11,22817) = 18.14, p <0.001		
*R*^*2*^ *total*	0.321		<0.001	0.312		<0.001	0.116		<0.001	0.150		<0.001
*Nutrition*												
Fruits	–0.025	0.022	0.251	–0.000	0.013	0.998	–0.013	0.009	0.127	–0.013	0.006	0.036
Vegetables	–0.050	0.021	0.017	–0.022	0.013	0.092	–0.020	0.008	0.014	–0.008	0.006	0.182
Hot meal	–0.187	0.037	<0.001	–0.050	0.023	0.029	–0.047	0.015	0.001	–0.088	0.011	<0.001
Breakfast	–0.041	0.029	0.155	–0.022	0.018	0.219	–0.014	0.011	0.220	–0.005	0.009	0.594
*ΔR*^*2*^	0.018 F(4, 5161) = 32.55, p <0.001			0.008 F(4, 5594) = 14.10, p <0.001			0.010 F(4, 6009) = 17.54, p <0.001			0.026 F(4, 5805) = 46.17, p <0.001		
	0.008 F(4, 3774) = 11.73, p <0.001			0.002 F(4, 4003) = 2.94, p = 0.019			0.006 F(4, 4204) = 7.25, p <0.001			0.018 F(4, 4144) = 22.19, p <0.001		
*R*^*2*^ *total*	0.346		<0.001	0.333		<0.001	0.150		<0.001	0.169		<0.001

^1^ Estimates of unstandardized regression coefficients (B) with standard error (SE) of background, drinking, smoking, and physical activity variables are based on the final regression model including all these predictors simultaneously, applied to both the West-Brabant (WB) and Hart voor Brabant, Brabant Zuidoost, Zeeland (HZ) together. *ΔR*^*2*^ is the increase in explained variance of adding all predictors of this block (drinking, smoking, physical activity) after controlling for the effect of the background variables (first line) or after controlling for all other predictors in the final model (last line).

Estimates of regression coefficients of nutrition are based on the model, including all predictors simultaneously for the WB sample. *ΔR*^*2*^ is here the increase in explained variance of adding all predictors of nutrition after controlling for the effect of the background variables (first line) or after controlling for all other predictors in the model (last line).

*‡* We did not include marital status in the regression analysis with frailty total and frailty social as dependent variables because living alone is a component of these variables

Not surprisingly, as almost half of total frailty comprises of physical frailty items, the results of the regression analyses on physical frailty were similar to those of total frailty (third column of Table 3). Two noteworthy results were that being a widow was associated to more physical frailty after controlling for other effects, and detrimental effects of smoking on physical frailty were very small (*f*^*2*^ = 0.004 and *ΔR*^*2*^ = 0.001 after only controlling for background variables and after controlling for all effects, respectively). Not surprisingly, the effect of the physical activity variables on physical frailty was much larger (*f*^*2*^ = 0.119 and *ΔR*^*2*^ = 0.074 when not controlling and after controlling for other effects, respectively).

Concerning the regression results of psychological and social frailty, we found considerably lower total explained variances for the psychological (*R*^*2*^ = 0.116) and social frailty (*R*^*2*^ = 0.150) than for physical frailty. And all lifestyle factors together also explained considerably less variance, after controlling for the background variables (*ΔR*^*2*^ = 0.116– 0.088 = 0.028 for psychological, and *ΔR*^*2*^ = 0.022 for social frailty). The smoking variables had very small detrimental effects on psychological (*f*^2^ = 0.003 and *ΔR*^*2*^ = 0.002) and small effects on social frailty (*f*^2^ = 0.010 and *ΔR*^*2*^ = 0.007). The drinking variables had very small beneficial effects on both psychological (*f*^2^ = 0.001 and *ΔR*^*2*^ = 0.001) and social frailty (*f*^2^ = 0.003 and *ΔR*^*2*^ = 0.003), but only how often one drank had a beneficial effect and not if one drank in the past. The physical activity variables had small beneficial effects of both frailty domains (*f*^2^ = 0.021 and *ΔR*^*2*^ = 0.016 for psychological, and *f*^2^ = 0.012 and *ΔR*^*2*^ = 0.007 for social), but these beneficial effects were only for light homework, cycling, walking, gardening, do-it-yourself, and sports.

Lifestyle factor nutrition (bottom of Table 3) had a small beneficial effect on total frailty (*f*^2^ = 0.02 and *ΔR*^*2*^ = 0.008), most attributable to its beneficial effect on social frailty (*f*^2^ = 0.032 and *ΔR*^*2*^ = 0.018) and less so on physical (*f*^2^ = 0.01 and *ΔR*^*2*^ = 0.002) and psychological frailty (*f*^2^ = 0.012 and *ΔR*^*2*^ = 0.006). Interestingly, only hot meal had a beneficial effect on all frailty domains after controlling for the other predictors, but not the intake of fruits or vegetables, or having breakfast.

## Discussion

Previous studies have shown that having an unhealthy lifestyle is associated with frailty [16–18]. Studies focused on specific lifestyle factors as smoking [20], excessive alcohol use [24], poor dietary habits [26], and low physical activity [29], provided evidence that these individual factors had an effect on frailty. However, frailty was predominantly defined as a biological concept, consisting of physical limitations that older people may have, mostly represented by the phenotype of frailty [3]. An added value of our study is that we used a broad definition of frailty. We aimed to determine cross-sectional associations between four lifestyle factors (smoking, alcohol use, nutrition, physical activity), and multidimensional frailty (physical, psychological, social) in large samples of Dutch community-dwelling older people aged 65 years or older, 10,421 and 34,915 individuals, respectively.

Using correlations, our study showed that all four lifestyle factors were associated with frailty total. A remarkable finding is that older people that smoked in the past were less frail, although this association was very small. Our first finding is not supported by the systematic review carried out by Kojima et al. [20]. However, we did not investigate how long it has been since people smoked and for how long. Moreover, our second variable “smoking” demonstrated that higher cigarette use was associated with more frailty. Most importantly, as correlations do not take effects of other predictors into account, this unexpected correlation is likely spurious. Indeed, after controlling for other predictors, smoking (past and present) was (albeit weakly) associated with more frailty.

Another rather unexpected finding is that both drinking in the past and a high number of days drinking were associated with less frailty, after controlling for demographic variables and other lifestyle factors, although these associations were small. As mentioned in the introduction, previous studies do not provide an unambiguous picture of the correlation between alcohol use and frailty [22–24]. Additionally, the association between alcohol consumption and health is still somewhat controversial, while it has become clear that heavy alcohol consumption is associated with poorer health, moderate alcohol consumption may or may not be associated with poorer health [40–42]. This possible nonlinear association between alcohol consumption and frailty is not captured by number of days of drinking alcohol. More studies are clearly warranted that focus on the negative association between alcohol consumption and frailty, and how this association relates to health outcomes.

Finally, as expected, many correlations between the physical activity variables and total frailty were higher compared with the associations of the other lifestyle factors with total frailty. This is mostly due to the fact that eight of the fifteen items of the TFI relate to the physical functioning of older people.

The regression analyses demonstrated that higher scores on alcohol use, physical activity, and nutrition, and lower scores on smoking were associated with less total, physical, psychological and social frailty, after controlling for all the prediction variables in the model, including sociodemographic characteristics of the participants (age, gender, marital status, education, income). The effects of smoking were small, but it is well known that smoking is very harmful to people's physical health. Smoking can be considered a strong risk factor for premature death [43]. Our study showed that smoking not only negatively influences physical frailty, but also the psychological and social frailty of older people, including feeling down, anxiety and lack of social relations. An explanation might be that smoking may lead to depression or anxiety, through effects on a person’s neurocircuitry that increases susceptibility to stressors in the environment [44], whereas smoking may effect loneliness as smoking is becoming more unacceptable in company with others. These frailty items are also closely related to items on the quality of life of older people [45]. These findings are also in line with Mesquita et al. [46] who found a direct association between smoking and worse scores on the mental health summary of the health-related quality of life Short Form Health Survey (SF-36) questionnaire in physically independent older people.

Not surprisingly, the effects of all physical activity variables together on physical frailty were high. Nine of eleven variables of physical activity were associated with physical frailty, underlining the importance of being physical active at an advanced age. A longitudinal cohort study using samples of community-dwelling older people in Greece, Croatia, the United Kingdom, Spain, and the Netherlands, showed that both maintaining a regular frequency and increasing to a regular frequency of physical activity were associated with lower physical frailty, along with lower psychological and social frailty, assessed with the TFI [47]. Currently, more and more studies are being carried out to determine the effect of a physical activity intervention on frailty. A recent systematic review and meta-analysis including 24 randomized controlled trials and two observational studies observed some evidence that various physical activity interventions are beneficial for frail older people [48]. Apóstolo et al. [10] concluded that physical exercise programs were only effective for reducing or postponing frailty if the programs were conducted in groups.

In the sample West-Brabant (WB) we showed that nutrition, including the variables hot meal, fruits, vegetables, breakfast, had a larger effect on social frailty than on physical and psychological frailty. This difference is mainly explained by the variable hot meal, although all four nutrition variables were (albeit weakly) associated to less total frailty when not controlling for the other predictors. Community-dwelling older people eat more varied food when someone is present. Preparing a hot meal can be a problem or be experienced as a nuisance if you are alone or feel lonely; living alone and loneliness are two of the three components of the social subscale of the TFI [32]. In the Kashiwa Study involving individuals aged ≥65 years, it was confirmed that eating alone was associated with frailty, assessed with the Kihon checklist [49]. A systematic review of longitudinal studies focusing on the associations between lifestyle factors and frailty showed that the associations between frailty and smoking were ambiguous [50]. However, negative associations were observed between frailty and higher consumption of fruit, vegetables, and alcohol. This review reflects an increasing attention towards modifiable risk factors for frailty which can be changed using behavioral interventions.

Many studies on the associations between lifestyle factors and frailty employ the phenotype of frailty by Fried et al [3], which only assesses the physical domain of frailty. In our study, both the correlations and the regression analyses showed that lifestyle factors were not only associated with physical frailty, but also with psychological and social frailty. Moreover, associations of lifestyle factors with frailty varied across domains, for instance, nutrition is most associated to the social domain, where physical activity is mostly associated to the physical domain. These findings emphasizes the importance of assessing frailty broadly and thus paying attention to the multidimensional nature of this concept. This statement is also supported by the fact that multidimensional frailty is predictive for disability, indicators of healthcare utilization (e.g., receiving personal care and nursing), and lower quality of life [32, 33, 45]. In this context, it is relevant to note that we used the TFI to measure multidimensional frailty and the three separate domains. Other operationalizations of physical, psychological, and social frailty exist [51]. Although we have no reasons to suspect that associations between lifestyle factors and frailty domains significantly depend on the assessment of these domain, we recommend conducting studies on the associations between lifestyle and frailty using other instruments than the TFI.

Our findings show that the demographic variables strongly affect total frailty and the frailty domains, and more strongly than the lifestyle factors, as is demonstrated by higher explained variances of frailty. Higher age, being a woman, and struggling with income were associated with frailty total and the three domains. These findings are in line with several studies among community-dwelling older people [1, 13, 15]. In the present study, we assessed income subjectively, but in largely the same Dutch sample using the TFI and an objective measure of income (net monthly income in euros) a similar finding was observed [13].

Our study had a number of limitations to which we must draw attention. First, there were many missing values concerning frailty total (n = 11,887) and lifestyle factors (e.g., 11,992 missing values were present with regard to active work light and active work heavy). Secondly, the assessment of lifestyle factors with our survey was relatively superficial with a limited number of questions. The smoking questions did not address how much one smoked and smokes, and the drinking questions how much one drank or drinks. The data with regard to the physical activity questions contained unlikely answers that we had to remove.

Thirdly, chronic diseases are known to have a strong association with multidimensional frailty, assessed with the TFI [52], but chronic diseases was not controlled for in the analyses reported in this study. We chose not to include it in our analyses as chronic diseases was strongly correlated to physical and total frailty (*r* close to .5), and we consider chronic diseases as another outcome of life style factors. However, we did also carry out all analyses in Table 3 with chronic diseases as a background variable; this did not affect our main conclusions but increased the total explained variances of total frailty and physical frailty with 10% and 13%, respectively.

Finally, the cross-sectional design of the study does not allow strict cause-effect interpretations between the four lifestyle factors and the frailty variables. Therefore, we recommend conducting a longitudinal study examining the effects of lifestyle factors on frailty in the short (1 year) and long term (10 years). In addition, we propose to carry out an intervention study including one or more lifestyle factors with multidimensional frailty as the primary outcome. For these future studies we also recommend more and better measures of lifestyle factors, ideally not based on self-report but on objective measures of behavior.

### Conclusions

In conclusion, our study showed that four lifestyle factors (smoking, alcohol use, physical activity, nutrition) were associated with multidimensional frailty, consisting of a physical, psychological, and social domain. The different associations of these frailty domains with lifestyle factors emphasize the importance of assessing frailty broadly and thus to pay attention to the multidimensional nature of this concept. Our findings offer healthcare professionals starting points for interventions with the purpose to prevent or delay the onset of frailty, so community-dwelling older people have the possibility to aging in place accompanied by a good quality of life. Aging in place fits well with the wish of many older people to stay in their own homes for as long as possible.

## Data Availability

The data that support the findings of this study are available from Dutch Health Services of the provinces Zeeland and Brabant (the Netherlands) but restrictions apply to the availability of these data, which were used under license for the current study, and so are not publicly available. Data are however available from the authors upon reasonable request and with permission of Dutch Health Services of the provinces Zeeland and Brabant (the Netherlands).

## Abbreviations

HZ: Hart voor Brabant, Brabant Zuidoost, Zeeland
SD: Standard deviation
SF-36: Short Form Health Survey
TFI: Tilburg Frailty Indicator
WB: West-Brabant
WHO: World Health Organization
